# *Astragalus membranaceus*—*Salvia miltiorrhiza* Decoction Ameliorates Metabolic Syndrome and Gut Microbiota Dysbiosis Induced by High‐Fat Diet: A Comparative Study

**DOI:** 10.1002/fsn3.71436

**Published:** 2026-01-09

**Authors:** Haiyin Zhang, Haofang Wan, Yihang Lu, Yu He, Haitong Wan, Chang Li

**Affiliations:** ^1^ Zhejiang Chinese Medical University Hangzhou China; ^2^ Zhejiang Key Laboratory of Chinese Medicine for Cardiovascular and Cerebrovascular Disease Hangzhou China; ^3^ Academy of Chinese Medical Sciences, Henan University of Chinese Medicine Zhengzhou China

**Keywords:** *Astragalus membranaceus* (Fisch.) Bunge, gut microbiota, high‐fat diet, metabolic syndrome, *Salvia miltiorrhiza* Bunge, short chain fatty acid

## Abstract

*Astragalus membranaceus* (Fisch.) Bunge (AM)— *Salvia miltiorrhiza* Bunge (SM) decoction (ASD) is a herb pair not only widely used in clinic against cardiovascular diseases and metabolic syndrome, but also as key compositions in some functional foods. However, the pairing ratios and potential mechanisms of ASD in preventing metabolic syndrome are still unclear. The present study comprehensively evaluated how the pairing ratios of AM and SM affect the treatment of ASD against high‐fat diet (HFD)‐induced metabolic disorder and investigate the roles of gut microbiota in the biological process. To establish a metabolic syndrome model, thirty SD male rats received a 10‐week HFD. All rats were divided into six groups randomly: ND, HFD, ASD‐A (the quality ratio of 1:2 AM to SM), ASD‐B (the quality ratio of 1:1 AM to SM), ASD‐C (the quality ratio of 2:1 AM to SM), and Simvastatin groups, and treated with corresponding medicine or saline. Biochemical changes and inflammation levels in serum and liver were assessed, while histopathology staining was conducted to evaluate liver and intestinal barrier lesions. Moreover, 16S rRNA sequencing technology was employed to assess the gut microbiota structure, and a targeted metabolomics method was utilized to quantify the fecal content of BAs and SCFAs. Administration of ASDs in three different pairing ratios considerably alleviated obesity, hyperlipidemia, systemic inflammation, liver injury, and intestinal barrier disorder in rats. Among the three decoctions, ASD‐A showed the greatest potential in multi‐aspects. Furthermore, ASD‐A significantly ameliorated gut microbiota dysbiosis and the imbalance of SCFAs and BAs induced by HFD. Especially, correlation analysis results suggested that the levels of lactic acid, 3‐hydroxybutyric acid, and 3‐hydroxypropionic acid are potential therapeutic biomarkers. The research findings indicated that ASDs could effectively prevent HFD‐induced metabolic syndrome, among which ASD‐A, with a quality ratio of 1:2 (AM: SM) exhibited superior effects. ASD‐A also significantly ameliorated gut microbiota disorders induced by HFD and promoted the production of beneficial metabolites.

## Graphical abstract

The optimal pairing ratio of ASDs against hyperlipidemia was systematically investigated. ASD maintains intestinal homeostasis and improves the systemic inflammatory response. A new LC–MS/MS method for the determination of derivatized SCFAs was established. ASD regulates the gut microbiota and its metabolites (SCFAs and BAs). Potential biomarkers of ASD against metabolic syndrome were proposed.

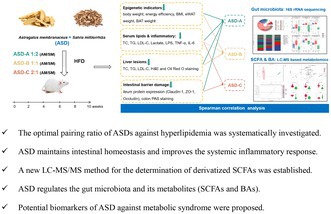

**Abbreviations**
2‐HB: 2‐hydroxybutyric acid
3‐HB: 3‐hydroxybutyric acid
3‐HP: 3‐hydroxypropionic acid
4‐BNMA: 4‐bromo‐N‐methylbenzylamine
AA: acetic acid
AM: *Astragalus membranaceus* (Fisch.) Bunge
ANOVA: analysis of variance
ASCVD: atherosclerotic cardiovascular disease
ASD: *Astragalus membranaceus* (Fisch.) Bunge—*Salvia miltiorrhiza* Bunge decoction
ASD‐A: ASD extract at a quality ratio of 1:2 (AM: SM)
ASD‐B: ASD extract at a quality ratio of 1:1 (AM: SM)
ASD‐C: ASD extract at a quality ratio of 2:1 (AM: SM)
ASVs: amplicon sequence variants
BA: butyric acid
BAs: bile acids
BAT: brown adipose tissue
BMI: body mass index
CTAB: cetyl trimethylamine bromide
CTT: cholesterol treatment trialists
EDC: 1‐ethyl‐3‐dimethylaminopropyl carbodiimide
eWAT: epididymal white adipose tissue
F/B: Firmicutes/Bacteroidetes
HA: hexanoic acid
HA‐d11: hexanoic acid‐D11
HDL‐C: high density lipoprotein cholesterol
H&E: hematoxylin and eosin
HFD: high fat diet
HMG‐CoA: 3‐hydroxy‐3‐methyl glutaryl coenzyme A
HPLC: high performance liquid chromatography
IL‐6: interleukin‐6
LA: lactic acid
LDA: linear discriminant analysis
LDL‐C: low density lipoprotein cholesterol
LEfSe: linear discriminant analysis effect size
L‐LA‐13C3: l‐lactic acid‐13C3 sodium
LPS: lipopolysaccharide
MRM: multiple reaction monitoring
ND: normal diet
PA: propionic acid
PA‐d5: propionic acid‐D5
PAS: periodic acid‐Schiff
PCoA: principal coordinate analysis
PCSK9: protein convertase subtilisin/kexin type 9
SA: succinic acid
SA‐d4: succinic‐D4
SCFA: short‐chain fatty acid
SD: sprague‐dawley
Simv: simvastatin
SM: *Salvia miltiorrhiza* Bunge
SPF: specific‐pathogen‐free
TC: total cholesterol
TCM: traditional Chinese medicine
TG: triglyceride
TNF‐α: tumor necrosis factor‐α
VA: valeric acid

## 1 Introduction

High‐fat diet is included in the major risk factors for cardiovascular‐related chronic diseases. Overnutrition and reduced energy expenditure can result in overweight or obesity, which is strongly related to metabolic diseases including atherosclerosis, diabetes mellitus, insulin resistance, and metabolic dysfunction‐associated fatty liver disease (J. Chen et al. 2023).

Many relevant publications have proven that there are strong associations between gut microbiota dysbiosis and hyperlipidemia. Under physiological conditions, gut microbiota has a symbiotic relationship with the host, which is crucial for maintaining the balance within the intestinal tract. However, the intestinal environment can be changed by lipid metabolism disorders, resulting in alterations in gut microbiota (Geng et al. 2022). Previous research has indicated that HFD can downregulate the relative abundance of *Bacteroidetes* and upregulate the relative abundance of *Firmicutes*, thus increasing the *Firmicutes*/*Bacteroidetes* (*F*/*B*) ratio (Stojanov et al. 2020). Metabolic syndrome was also reported to be associated with the decline of probiotics, such as *Prevotella*, *Ackermannia*, and *Bifidobacterium*. In addition, there is growing evidence that metabolites such as SCFAs, BAs, and branched‐chain amino acids, which are closely related to gut microbiota, play vital roles in preventing and treating metabolic syndromes (Huang et al. 2019).

Dyslipidemia is characterized by increased serum TC, TG, and LDL‐C levels and decreased HDL‐C level. Especially, clinical and genetic researches have revealed that increased LDL‐C levels are linked to higher ASCVD risk (Ference et al. 2017). The administration of statins is a vital contemporary strategy for reducing morbidity and mortality from atherosclerotic vascular diseases. However, in 2010 the CTT meta‐analysis, which included individual‐level data from 26 randomized controlled trials of statin therapy, it was found that every 0.5 mmol/L reduction in LDL‐C concentrations might lead to a 15% reduction in the incidence of major vascular events (Baigent et al. 2010). However, statin medications, while effective, are frequently discontinued owing to adverse reactions (Cohen et al. 2012). Previous reports demonstrated that patients who are unable to tolerate statins experience a 36% higher incidence of recurrent myocardial infarction and a 43% greater rate of coronary events than those who adhere well to statin regimens (Serban et al. 2017). These findings implied that statin intolerance is closely related to a greater risk of relapsing heart attack and coronary heart disease. Ezetimibe and bile acid sequestrants serve as effective alternatives for lowering LDL‐C levels via downregulation of intestinal cholesterol absorption. These drugs, however, can also increase gastrointestinal adverse effects and interfere with the absorption of other medications (Xie et al. 2012). PCSK9 inhibitors (e.g., Evolocumab) enhance the LDL‐C receptors count in the liver by mediating degradation of the LDL‐C receptor, thereby lowering circulating LDL‐C levels, while PCSK9 inhibitors can lead to nasopharyngitis, upper respiratory tract infections, and other adverse reactions (Raal et al. 2015).

As described in “Qian Jin Yi Fang” which is written in the 7th century, ASD was useful in the treatment of consumptive diseases. Both AM and SM are not only used as herbal medicine, but also as functional food and tonic tea in some Asian countries for decades. Modern research has validated their antioxidant, anti‐inflammatory, and endothelial‐protective properties, which support their incorporation into health‐promoting food matrices (Zhou et al. 2005). Recent developments included the formulation of AM and SM containing teas and other functional beverages, together with products such as yogurts and cereal foods, in which AM and SM extracts were added as nutritional additives. There are also technological progresses related to AM and SM in food sciences, especially the application of new encapsulation technologies such as nanoemulsions and liposomes, to enhance the stability and bioavailability of the major bioactive components in the decoction during processing and gastrointestinal transit (Zhou et al. 2005). AM and SM frequently appear as key compositions of nutritional food supplements with significant health benefits. For example, the functional food JRP‐SNF102, which includes a combination of AM, *Trichosanthes kirilowii*, and *Angelica gigas*, has been shown to effectively reduce inflammatory skin responses (Han et al. 2023). Furthermore, the combination of AM and *Panax notoginseng* was recognized by the Food Safety Authority of European Union in 2020 as a new food supplement enhancing intestinal nutrient absorption (EFSA Panel on Nutrition Novel Foods and Food Allergens (NDA), et al. 2020). SM was also reported as a dietary supplement to facilitate vasodilation and improve microcirculation. Moreover, natural polyphenols derived from SM inhibited NLRP3 inflammasomes, thereby offering potential therapeutic benefits in the prevention of inflammatory and metabolic diseases (Wang et al. 2023). AM and SM were both representative drug pairs for promoting blood circulation and nourishing *qi* in the theory of TCM, which have been reported to improve myocardial remodeling and cardiac dysfunction with modern pharmacological methods (Chen et al. 2024; Xu et al. 2024). Studies have shown that administration of AM extract leads to a decrease in the mass of both hepatic and adipose tissues, regulating serum and liver lipid levels in non‐alcoholic liver disease mice (Zheng et al. 2024). SM extracts have also been reported to significantly reduce body mass and body fat index, modulate blood lipid levels, and ameliorate lipid metabolism disorders in HFD‐induced rats (Ai et al. 2022). Some of the widely used compound TCM prescriptions containing ASD, Naoxintong capsule, for example, exhibited great potential in the treatment of early‐stage atherosclerosis and hyperlipidemia (Lu et al. 2022; Wan et al. 2024).

Most of the published investigations focused on one special pre‐designed ratio of AM and SM (usually 2:1) to explore their effects (Han et al. 2021; Shen et al. 2023). In the present work, three different ratios of ASD, namely 1:2 (ASD‐A), 1:1 (ASD‐B), and 2:1 (ASD‐C), were evaluated in comparison. By comprehensively comparing the effects of these three ratios on HFD‐induced obesity, metabolic abnormalities, liver and intestinal barrier damage, the optimal ratio of AM and SM to ameliorate metabolic syndrome was identified. Especially, the study focused on the influences of ASDs on gut microbiota, which indirectly affect metabolic syndrome.

## 2 Materials and Methods

### 2.1 Reagents

AA (98%), PA (98%), BA (98%), VA (98%), HA (98%), SA (98%), PA‐*d*_5_ (98%), and SA‐*d*_*4*_ (98%), and the SCFA derivatization agents (4‐BNMA and EDC, 98%) were purchased from Shanghai Aladdin Biochemical Technology. HA‐*d*_11_ (98%) was purchased from Shanghai Macklin Biochemical Technology. LA (85%–90%), 3‐HP (95%), 2‐HB (95%), 3‐HB (95%), and Simv (98%) were purchased from Shanghai Yien Chemical Technology. L‐LA‐^13^C_3_ (200 mg/mL) was purchased from MedChemExpress (New Jersey, USA). Acetonitrile and methanol were purchased from Tedia (HPLC‐grade, Hefei, China). Ultrapure level water was obtained from Milli‐Q instrument. The D12450H (ND) and D12451 (HFD) feed were purchased from Jiangsu Xietong Pharmaceutical Bio‐Engineering.

### 2.2 Preparation and Quality Control of ASDs

AM (Batch No. 2105021126) was purchased from Linshi Shengtai Pharmaceutical and SM (Batch No. WL231225) was purchased from Wanli Chinese Herbal Medicine. Both AM and SM materials were identified with reference to the Chinese Pharmacopeia 2020. The two herbs were powdered and sieved through a 60‐mesh sieve, and a certain amount of the two herbs was weighed according to the quality ratio of 1:2 (ASD‐A), 1:1 (ASD‐B), and 2:1 (ASD‐C) AM to SM. Ten times the volume of water was added and soaked overnight. Heated reflux at 100°C was then performed twice. After combining the two filtrates, the solution was concentrated to 4.2 g/mL. Quality control of the ASDs was conducted with HPLC. The extracts were diluted 100 times before conducting qualitative and quantitative analysis by HPLC on Ultimate XB‐C18 (150 mm × 4 mm, 3 μm) using Agilent 1260 Infinity II. The mobile phases were 0.1% formic acid (phase A) and acetonitrile–methanol (1:1, 0.1% formic acid, phase B). The injection volume was 10 μL, and the flow rate was 0.4 mL/min. The total polysaccharide contents in the ASDs were assessed based on a previous study (Ye et al. 2025).

### 2.3 Animals

Thirty‐six male SD rats (150 ± 20 g, SPF grade) were provided by Shanghai Slac Laboratory Animal. All rats were housed under standard temperature and humidity conditions with a 12 h light/dark cycle. All experiments were authorized by the Animal Experimentation Committee of Zhejiang Chinese Medicine University and carried out in accordance with its ethical guidelines (No. IACUC20220627‐13, registered on Jun. 27th, 2022).

Rats were acclimatized for 7 days and then randomized into six groups (*n* = 6): ND group: fed with normal diet; HFD group: fed with HFD; ASD‐A group: fed with HFD and ASD‐A (AM:SM = 1:2, 10.5 g/kg/d); ASD‐B group: fed with HFD and ASD‐B (AM:SM = 1:1, 10.5 g/kg/d); ASD‐C group: fed with HFD and ASD‐C (AM:SM = 2:1, 10.5 g/kg/d); Simv group: fed with HFD and Simv (10 mg/kg/d). During the ten‐week administration, rats were fed freely, and rats in the ND and HFD groups were treated with equal amounts of saline via gavage. Food consumption and body weight of the rats were recorded weekly. The calculation formulas for the relevant parameters are as previously reported (Li et al. 2024).

### 2.4 Sample Collection

Fresh feces were collected in sterile centrifuge tubes the day before the end point. Subsequently, all rats were fasted for 12 h and blood was collected under isoflurane anesthesia. After 0.5 h at room temperature, serum was obtained by centrifugation (3500 rpm, 10 min, 4°C). After cervical dislocation, the liver, epididymal fat, colon, and ileum were collected, and liver and eWAT were weighed. All samples were stored at −80°C prior to analysis.

### 2.5 Biochemical Analysis

The levels of TC, TG, LDL‐C, and HDL‐C in both serum and liver, and the levels of lactate in serum were determined with commercial kits (Nanjing Jiancheng Bioengineering Institute). Serum TNF‐α, IL‐6, and LPS levels were measured by ELISA kits (Elabscience and Jiyinmei).

### 2.6 Histopathological Analysis

After fixation in 4% paraformaldehyde for 24 h, the liver, colon, eWAT, and BAT were embedded in paraffin and sliced into 4‐μm thick sections. Subsequently, H&E staining was carried out on liver, eWAT, and BAT tissue sections, and H&E or PAS staining was performed on colon tissue sections. The liver tissue was embedded in OCT, quickly frozen, and sliced into 10‐μm thick sections. Subsequently, all liver tissue sections were stained with oil red O. All stained sections were evaluated under an Axio Observer Z1 microscope and an optical microscope equipped with a digital camera.

### 2.7 Immunohistochemistry

The tight junction proteins' expression was assessed using immunohistochemistry. In summary, ileal tissue embedded in paraffin was sliced into sections 4‐μm thick, which then underwent paraffin extraction, rehydration, and antigen retrieval processes. Subsequently, all sections were incubated overnight at 4°C with primary antibodies, including ZO‐1, Occludin, and Claudin‐1. This was followed by a 30‐min incubation with secondary antibodies at room temperature and the application of DAB for color development.

### 2.8 Determination of the Fecal Levels of SCFAs

The SCFAs in rat feces were derivatized using 4‐BNMA and then quantitatively analyzed using an AB SCIEX QTRAP 4500 mass spectrometer coupled with a Nexera X2 LC‐30 liquid chromatography. The detailed derivation procedure was provided in Appendix S1. The liquid chromatography conditions and mass spectrometry parameters were displayed in LC–MS parameter settings and Table S1.

### 2.9 Determination of the Fecal Levels of BAs

The fecal levels of 29 BAs were determined with a LC–MS based method. The measurements were carried out on a Q‐Exactive mass spectrometer (Thermo, San Jose, USA) coupled with U3000 UHPLC system (Thermo, San Jose, USA). The detailed method was shown in “Determination of the fecal levels of BAs” in Appendix S1. The mass spectrometry information for BAs and the internal standard was provided in Table S2.

### 2.10 Gut Microbiota Analysis

The 16S rRNA sequencing method was as described previously (Lu et al. 2022). The detailed procedures and methods are provided as ‘Gut microbiota analysis’ in Appendix S1.

### 2.11 Statistical Analysis

ANOVA was adopted to assess the statistical significance across groups. To verify statistical differences between groups, Dunnett's multiple comparison tests were conducted. The Kruskal–Wallis H test was utilized to confirm statistical differences among the three ASDs groups. GraphPad Prism 9 (GraphPad Software, San Diego, USA) was used for plotting histograms and line graphs. Differential metabolite analyses are performed in the Metaboanalyst platform (https://www.metaboanalyst.ca/).

## 3 Results and Discussion

### 3.1 Quantitation of Main Components in ASDs

**FIGURE 1:**
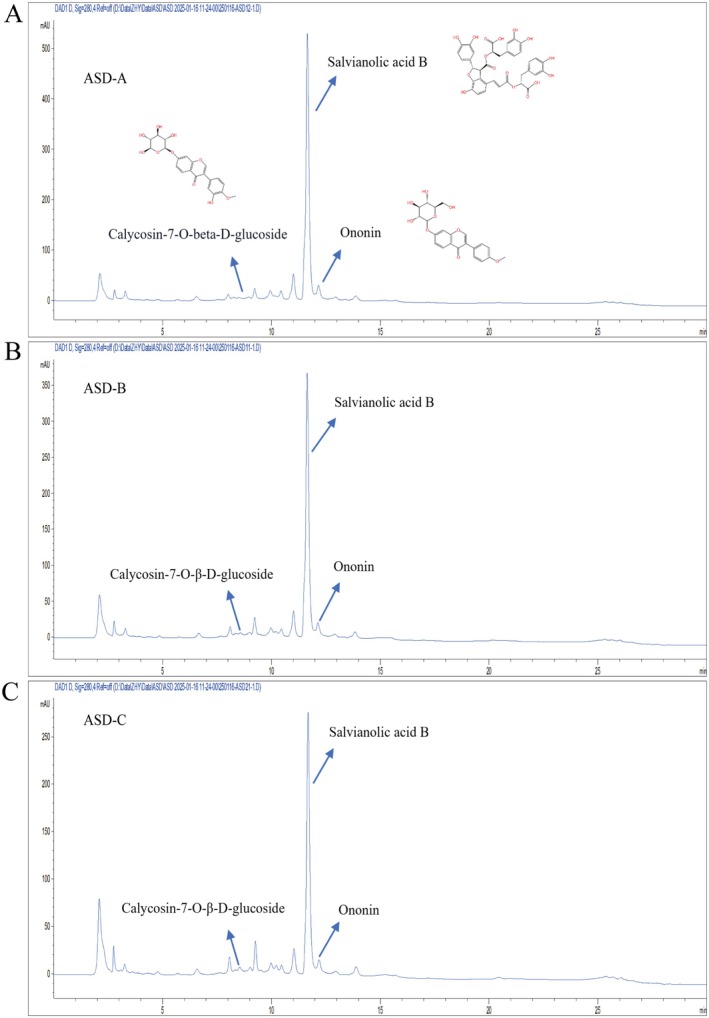
HPLC chromatograms of ASDs at three pairing ratios (wavelength = 280 nm). Peak annotation as shown.

Quantitation of aqueous extracts of ASDs with three different pairing ratios was conducted utilizing HPLC. As shown in Figure 1A–C, the three aqueous extracts contained three primary components: salvianolic acid B, ononin, and calycosin‐7‐O‐β‐D‐glucoside. These three components were subsequently subjected to quantitative analysis (refer to Table S3 for standard curves). The aqueous extract of AM was reported to contain flavonoids and saponins, including ononin, formononetin, calycosin, and calycosin‐7‐O‐β‐D‐glucoside (Shahrivari‐Baviloliaei et al. 2024). In the present study, as shown in Table 1, the concentrations of ononin in ASD‐A, ASD‐B, and ASD‐C were 0.32, 0.21, and 0.19 mg/g, respectively. The concentrations of calycosin‐7‐O‐β‐D‐glucoside were 0.01, 0.02, and 0.02 mg/g, respectively. The aqueous extract of SM mainly consist of hydrosoluble phenolic acids and few amounts of liposoluble tanshinones, with salvianolic acid B as the most prominent one (Yuen et al. 2021). In the present study, the concentrations of salvianolic acid B in ASD‐A, ASD‐B, and ASD‐C were determined to be 6.59, 4.78, and 3.35 mg/g. We further determined the contents of the total polysaccharides in the ASDs, and the results showed that the total polysaccharides contents in ASD‐A, ASD‐B, and ASD were 18.31, 14.75, and 15.86 mg/g, respectively.

**TABLE 1:** The content of main components in ASDs water extract.

Contens (mg/g)	ASD‐A	ASD‐B	ASD‐C
Salvianolic acid B	6.59	4.78	3.35
Ononin	0.32	0.21	0.19
Calycosin‐7‐O‐β‐D‐glucoside	0.01	0.02	0.02
Total polysaccharide	18.31	14.75	15.86

### 3.2 ASD Inhibits HFD‐Induced Weight Gain and Fat Accumulation

**FIGURE 2:**
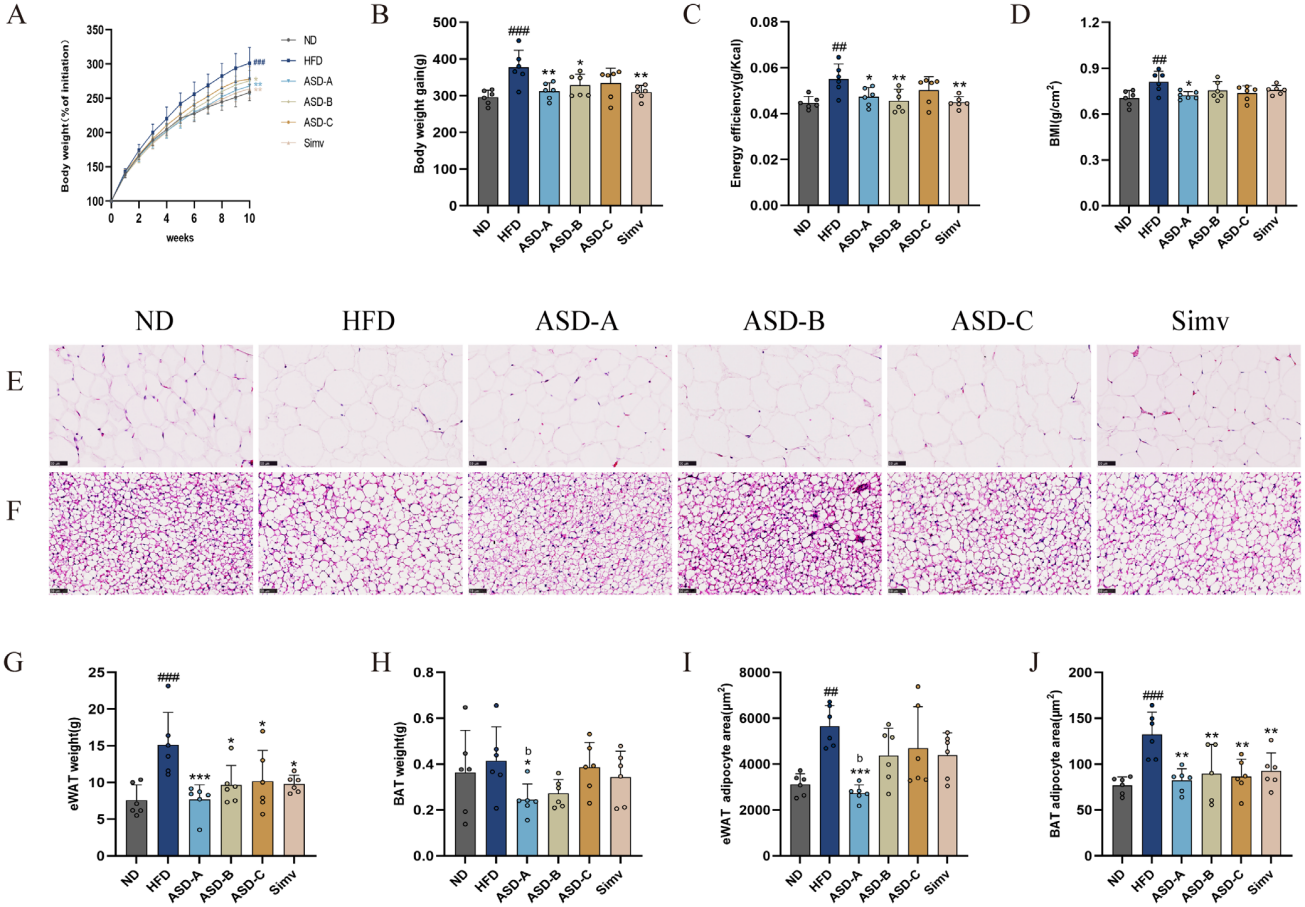
Effect of ASDs in varying rations on body weight, energy efficiency, tissue mass and histopathology of adipocytes in HFD‐induced obese rats. (A) Percentage of body weight; (B) Body weight gain; (C) Energy efficiency; (D) BMI; (E) H&E staining of eWAT and (F) BAT (bar = 50 μm, 400×); (G) eWAT mass; (H) BAT mass; (I) eWAT adipocyte area; (J) BAT adipocyte area. Data are expressed as mean ± SD (*n* = 6), ^##^ *p* < 0.01, ^###^ *p* < 0.001 compared with the ND group; *p < 0.05, **p < 0.01, ***p < 0.001 compared with the HFD group; ^b^ *p* < 0.05 for ASD‐A group vs. ASD‐C group.

The effects of ASD at the three ratios on HFD‐induced metabolic syndrome were investigated for a 10‐week period. As shown in Figure 2A, HFD resulted in a 16.5% increase in body weight in rats (*p* < 0.001), implying successful model establishment. HFD‐induced increases in body weight gain, energy efficiency, and BMI, ASD‐A remarkably lowered by 17.2%, 14.1%, and 11.1%, respectively. ASD‐B considerably decreased by 13% and 17.1% in body weight gain and energy efficiency of rats, respectively, whereas ASD‐C did not significantly alleviate these three indices in rats (Figure 2B–D). All the supplementations also significantly led to varying degrees of reduction in eWAT and BAT mass in rats, which partially accounted for the decrease in their body weight. Notably, comparing with the HFD group, ASD‐A considerably decreased eWAT mass by 49.5% and BAT mass by 41.1% in rats (Figure 2G,H). Moreover, HFD‐induced enlargement of adipocytes in the eWAT and BAT of rats was significantly inhibited by ASD‐A (*p* < 0.01), whereas ASD‐B and ASD‐C only inhibited the enlargement of adipocytes in the BAT (Figure 2E,F,I,J). Consequently, it is not difficult to find that ASD‐A was significantly more effective than ASD‐B and ASD‐C in mitigating adipose accumulation and restricting adipocyte enlargement in rats.

### 3.3 ASD Ameliorates HFD‐Induced Metabolic Disorders and Inflammation

**FIGURE 3:**
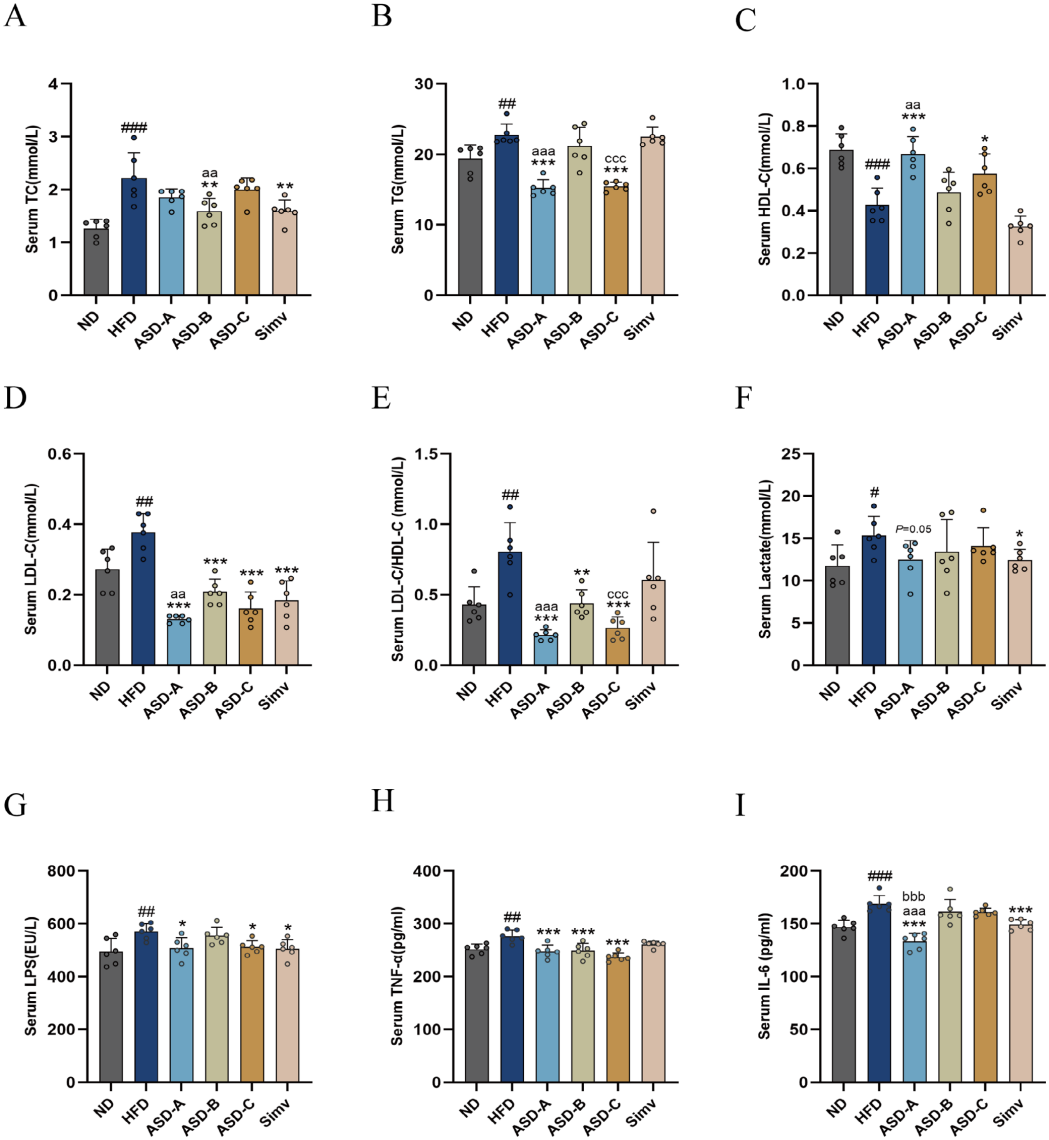
Effect of ASDs in varying ratios on serum levels of lipids, lactate, and inflammatory cytokine in HFD‐induced obese rats. (A) TC, (B) TG, (C) HDL‐C, (D) LDL‐C, (E) LDL‐C/HDL‐C, (F) Lactate, (G) LPS, (H) TNF‐α, and (I) IL‐6 in serum. Data are expressed as mean ± SD (*n* = 6). ^#^ *p* < 0.05, ^##^ *p* < 0.01, ^###^ *p* < 0.001 compared with the ND group; *p < 0.05, **p < 0.01, ***p < 0.001 compared with the HFD group; ^aa^ *p* < 0.01, ^aaa^ *p* < 0.001 for ASD‐A group vs. ASD‐B group; ^bbb^ *p* < 0.001 for ASD‐A group vs. ASD‐C group; ^ccc^ *p* < 0.001 for ASD‐B group vs. ASD‐C group.

Inflammation has an important influence on the onset and development of metabolic syndrome, which leads to metabolic dysfunction. HFD was reported as one of the most important ways to induce serum lipid metabolism disorders and inflammatory responses (Masenga et al. 2023). HFD results in increased intestinal permeability, which then promotes the absorption of exogenous free fatty acids into the circulatory system and their subsequent distribution to various organs and tissues. As depicted in Figure 3A–E, HFD considerably increased serum TC, TG, and LDL‐C levels in rats (*p* < 0.01), while reducing HDL‐C levels in rats (*p* < 0.001). Simv notably decreased serum TC and LDL‐C levels but had no noticeable impact on serum TG and HDL‐C levels in rats. ASD‐A and ASD‐C considerably decreased serum TG (33.1% and 32%, respectively) and LDL‐C levels in rats (65.4% and 57.1% respectively), while increasing HDL‐C levels in rats (66.7% and 34.8% respectively). Notably, ASD‐A outperformed ASD‐B in its effects on TG, LDL‐C, and HDL‐C levels in rats (*p <* 0.01), whereas ASD‐B only significantly improved serum TC levels in rats compared to ASD‐A (*p <* 0.01). Furthermore, prolonged HFD consumption typically results in inflammation and the dysregulation of adipocytokines. It has been reported that HFD induces adipocyte hypertrophy in rats, altering adipokine and pro‐inflammatory cytokine secretion, and promoting immune cell recruitment, especially macrophages, which are a prime source of pro‐inflammatory cytokines like TNF‐α and IL‐6. As illustrated in Figure 3F–I, HFD significantly increased serum lactate levels in rats, which were mitigated by the ASD‐A. In addition, in comparison with the ND group, HFD also induced a substantial 15.3% rise in serum LPS levels in rats, subsequently triggering significant increases in serum TNF‐α and IL‐6 levels by 9.8% and 14.9% in rats, respectively. ASD‐A markedly reduced serum LPS levels by 11.1%, TNF‐α levels by 10.4%, and IL‐6 levels by 21% in rats compared with those in the HFD group. In addition, ASD‐A exhibits a marked superiority over ASD‐B and ASD‐C in its capacity to improve IL‐6 levels in rats. These results indicate that ASD‐A exerts a more ameliorative effect on metabolism and cytokine disorders in rats than ASD‐B and ASD‐C.

### 3.4 ASD Ameliorates HFD‐Induced Hepatic Lipid Accumulation

**FIGURE 4:**
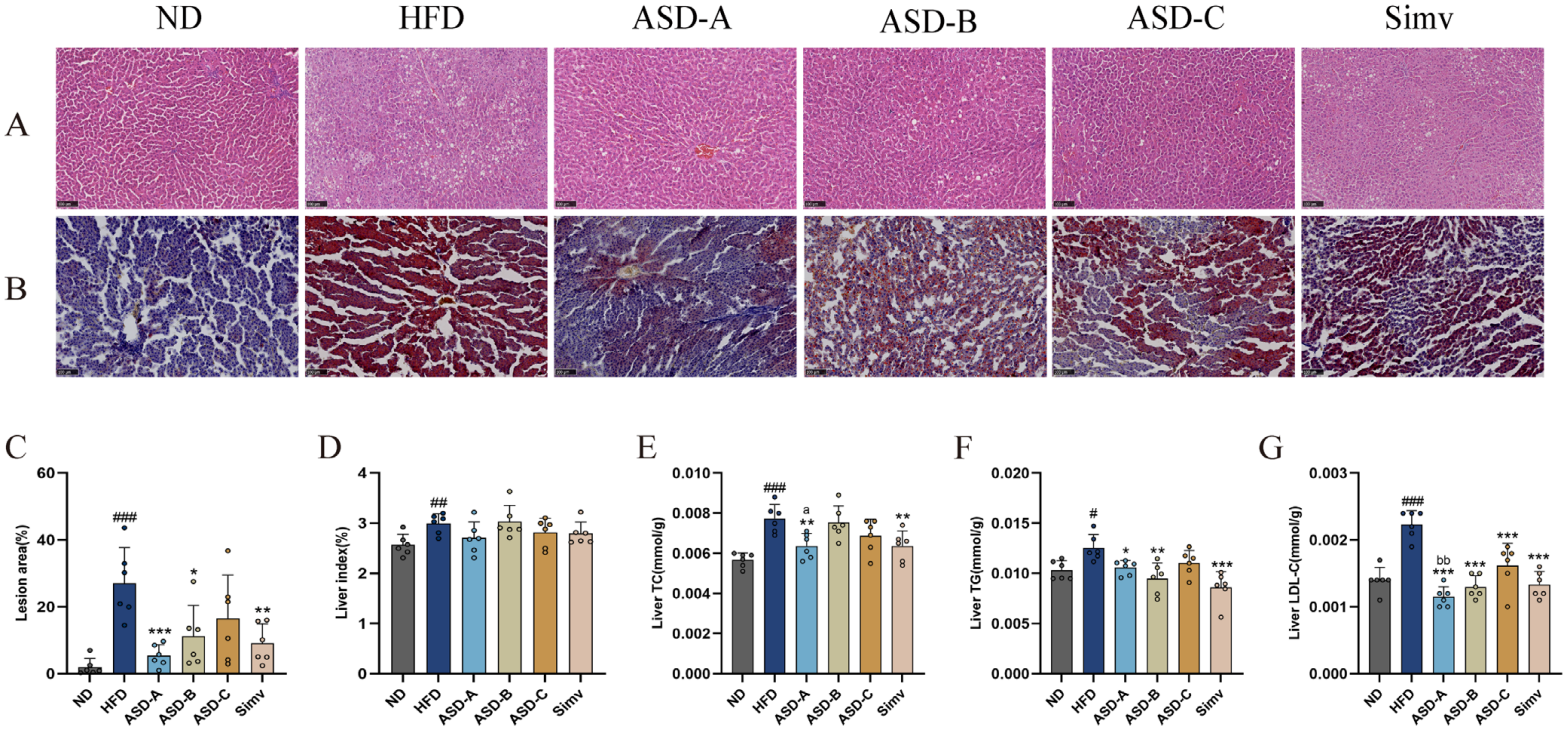
Effects of ASDs in varying ratios on hepatic steatosis of rats. (A) H&E staining of liver (bar = 100 μm, 200×); (B) Oil red O staining of liver (bar = 100 μm, 200×); (C) Quantitative analysis of oil red O staining of liver; (D) Hepatic index; (E) Hepatic TC; (F) Hepatic TG; and (G) Hepatic LDL‐C. Data are expressed as mean ± SD (*n* = 6), ^#^ *p* < 0.05, ^##^ *p* < 0.01, ^###^ *p* < 0.001 compared with the ND group; *p < 0.05, **p < 0.01, ***p < 0.001 compared with the HFD group; ^a^ *p* < 0.05 for ASD‐A group vs. ASD‐B group; ^bb^ *p* < 0.01 for ASD‐A group versus ASD‐C group.

HFD significantly induced lipid accumulation, vacuolar degeneration, and cell hypertrophy in the liver of rats, which were notably improved by ASD‐A and ASD‐B, with ASD‐A showing the most significant improvement (*p* < 0.001, Figure 4A–C). Chronic HFD results in lipid metabolism disorders and hepatic fat accumulation. As shown in Figure 4D–G, HFD considerably elevated the liver index and lipid levels in rats. The increase in liver mass was inhibited to varying degrees in all supplementations, which partially accounted for the reduction in body weight in rats. Simv markedly reduced hepatic TC, TG, and LDL‐C levels in rats by respectively 18.1%, 31.4%, and 40.4% compared with the HFD group. ASD‐A resulted in a reduction in hepatic TC, TG, and LDL‐C levels in rats by 17.6%, 15.8%, and 48%, respectively, and demonstrated significantly greater effectiveness in ameliorating TC and LDL‐C levels than ASD‐B and ASD‐C (*p* < 0.05). Moreover, ASD‐B considerably decreased hepatic TG and LDL‐C levels (24.3% and 40.9%, respectively), which is similar to the effect of ASD‐A, but had no effect on hepatic TC levels in rats; ASD‐C only significantly reduced hepatic LDL‐C levels (26.9%) in rats. The results demonstrated that ASD‐A was more effective than ASD‐B and ASD‐C in ameliorating liver injury in rats, comparable to Simv.

### 3.5 ASD Protects Intestinal Barrier From HFD‐Induced Damage

**FIGURE 5:**
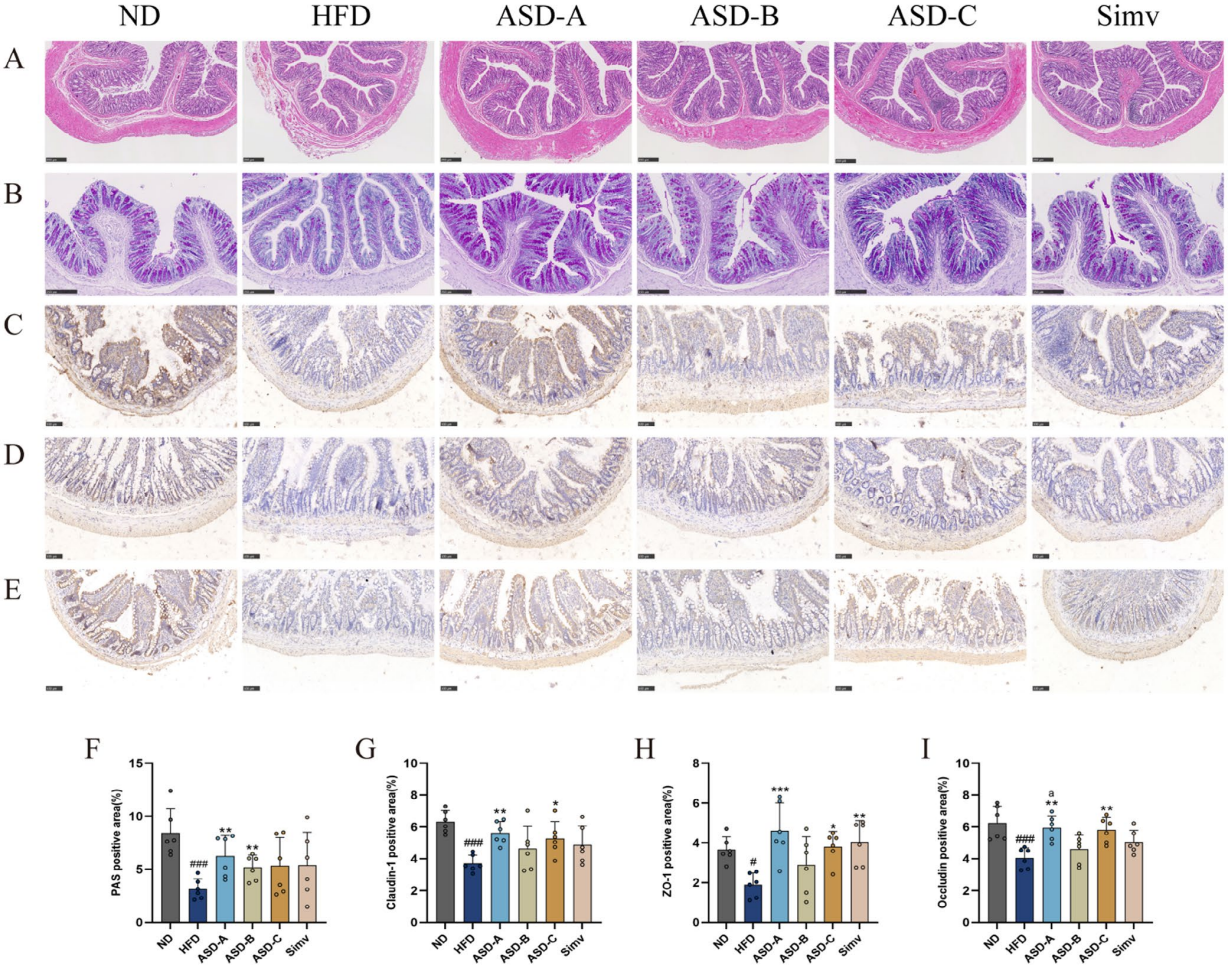
Effect of ASDs in varying ratios on intestinal barrier function and integrity of rats. (A) H&E staining of colon (bar = 250 μm, 100×); (B) PAS staining of colon (bar = 250 μm, 100×); Immunohistochemical staining for Claudin‐1 (C), ZO‐1 (D) and Occludin (E) in ileum (bar = 100 μm, 200×); (F) Quantitative analysis of PAS staining of colon; Quantitative analysis of immunohistochemical staining for Claudin‐1 (G), ZO‐1 (H) and Occludin (I) in ileum. Data are expressed as mean ± SD (*n* = 6), ^#^ *p* < 0.05, ^###^ *p* < 0.001 compared with the ND group; *p < 0.05, **p < 0.01, ***p < 0.001 compared with the HFD group; ^a^ *p* < 0.05 for ASD‐A group vs. ASD‐B group.

Long‐term HFD can also result in down‐regulation of tight junction proteins expression, which in turn destroys the intestinal physical barrier and increases the risk of various intestinal diseases in the host (Guo et al. 2020). As depicted in Figure 5A, HFD substantially caused deterioration of the muscular layer and mucosa, partial crypt loss, and inflammatory cell invasion in the colon of rats. All supplementations mitigated mucosal damage and crypt loss in colonic tissues without attenuating colonic inflammatory cell infiltration. HFD also resulted in a markable decrease in the goblet cell count and neutral mucin levels within the colon of rats (*p* < 0.001, Figure 5B,F), and ASD‐A and ASD‐B markedly reserved the damage by 98.1% and 64.3%, respectively. Chronic HFD has been reported to diminish the intestinal tight junction proteins expression and increase intestinal permeability, which results in increased intestinal permeation of LPS. As depicted in Figure 5G–I, the long‐term HFD considerably reduced the Claudin‐1, ZO‐1, and Occludin proteins expression in rats by 41.5%, 48.4%, and 35.3%, respectively, which may account for the elevated serum LPS levels in rats (Figure 3G). Simv only increased ZO‐1 protein expression in rats (Figure 5H). ASD‐A and ASD‐C markedly elevated the Claudin‐1, ZO‐1, and Occludin proteins expression in rats (Figure 5G,H). Notably, ASD‐B did not significantly increase protein expression in rats. These results indicated that ASD‐A and ASD‐C effectively mitigated HFD‐induced intestinal barrier damage in rats and demonstrate superior efficacy compared to Simv.

The results of the above studies showed that ASD‐A has a significant advantage in reducing BAT mass and adipocyte size, decreasing serum TG and LDL‐C levels, increasing HDL‐C levels, lowering serum lactate and IL‐6 levels, reducing hepatic TC and LDL‐C levels, and enhancing Occludin protein expression in rats. The ASD‐A water extract primarily consists of the total polysaccharides and salvianolic acid B (SAB) (Figure 1 and Table 1). Previous studies have demonstrated that SM polysaccharides and SAB have multiple pharmacological effects. SM polysaccharide has been found to inhibit inflammation and obesity through modulating the intestinal microbiota‐related gut‐liver axis (Lixia Li et al. 2022). While SAB can directly ameliorate HFD‐induced obesity and dyslipidemia. There are also many studies shown that SAB improves colonic inflammation and obesity via modulating gut microbiota (Lin Li et al. 2020). Thus, the fecal samples of ASD‐A were then subjected to further microbiomics and metabolites investigations.

### 3.6 ASD‐A Amended Gut Microbiota Dysbiosis in HFD‐Induced Rats

**FIGURE 6:**
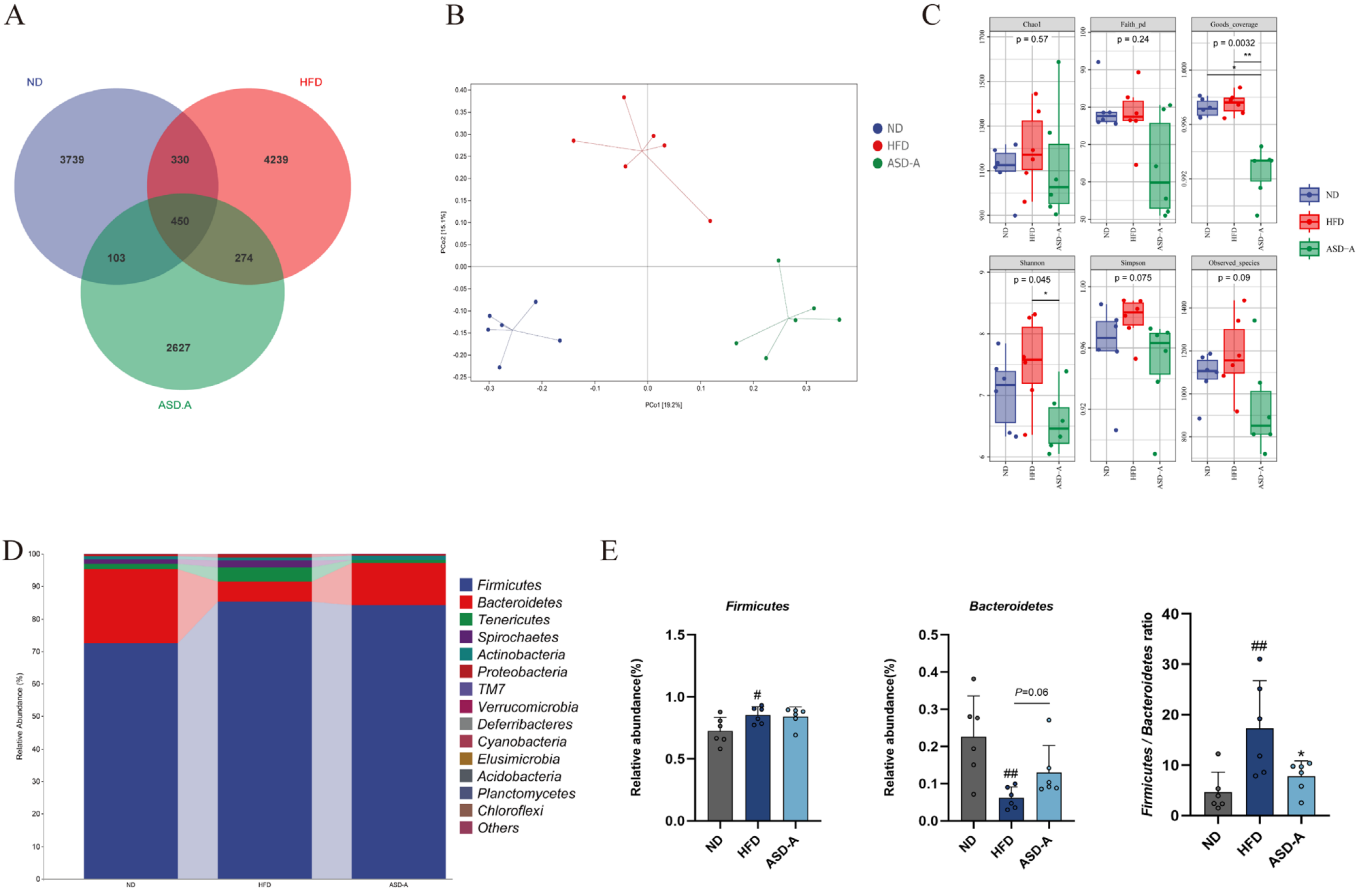
Modulation of ASD‐A on diversity and composition of gut microbiota in HFD‐induced obese rats. (A) Venn diagram; (B) PCoA plot; (C) α‐diversity index; (D) The relative abundance of species at phylum level (top 20); (E) The relative abundance of *Firmicutes*, *Bacteroidetes*, and F/B ratio (*n* = 6). Data are expressed as mean ± SD (*n* = 6), ^#^ *p* < 0.05, ^##^ *p* < 0.01 compared with the ND group; *p < 0.05 compared with the HFD group.

To examine the impact of ASD on the gut microbiota in rats, 16S rRNA sequencing was employed to evaluate the composition and abundance of gut microbiota within the ND, HFD, and ASD‐A groups. The Venn diagram showed that the OUT counts for the ND, HFD, and ASD‐A groups were 3739, 4239, and 2627, respectively (Figure 6A). The Bray‐Curtis distance revealed a distinct separation in the gut microbiota among the ND, HFD, and ASD‐A groups, indicating compositional differences in the β‐diversity among these three groups (Figure 6B). Alpha diversity analysis in the HFD group revealed that Chao1, Simpson, Good's coverage, Simpson, Observed species, and Faith PD indexes increased in different degrees compared with the ND group, whereas these parameters in the ASD‐A group showed a decreasing trend compared to the HFD group (Figure 6C). The *Firmicutes/Bacteroidetes* ratio is commonly used as an indicator of describing alterations in gut bacterial species. In animal and human models, the HFD‐driven microbiota composition changes are characterized by an elevated *Firmicutes/Bacteroidetes* ratio (Malesza et al. 2021). As depicted in Figure 6D,E, at the phylum level, *Firmicutes* is absolutely dominant, followed by *Bacteroidetes*. HFD increased the abundance of *Firmicutes* and decreased the relative abundance of *Bacteroidetes*, which was reflected in the significantly higher F/B ratio in the HFD group than in the ND group. ASD‐A significantly lowered the F/B ratio, despite not affecting the relative abundance of *Firmicutes*.

**FIGURE 7:**
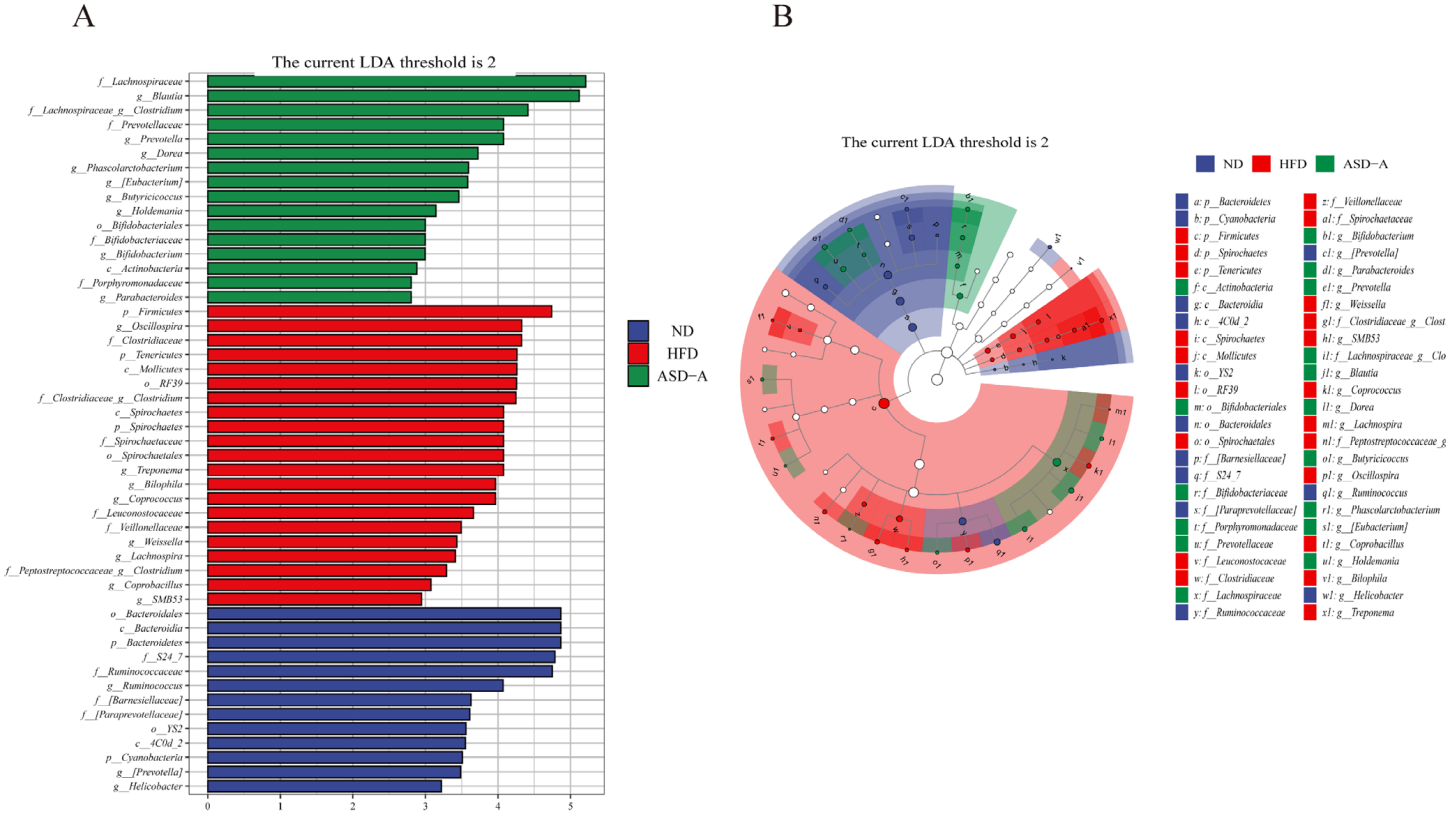
LEfSe analysis among ND, HFD and ASD‐A groups. (A) Histogram of LDA score based on LEfSe analysis (LDA > 2 was considered to be the differential characteristic taxon); (B) Taxonomic cladogram based on LEfSe analysis.

LEfSe analysis was used to search for biomarkers of metabolic syndrome in the gut microbiota among the three groups. Fifty differentially enriched taxa with an LDA score > 2.0 were identified, of which 13 were markedly enriched in the ND group, 21 in the HFD group, and 16 in the ASD‐A group (Figure 7A,B).

**FIGURE 8:**
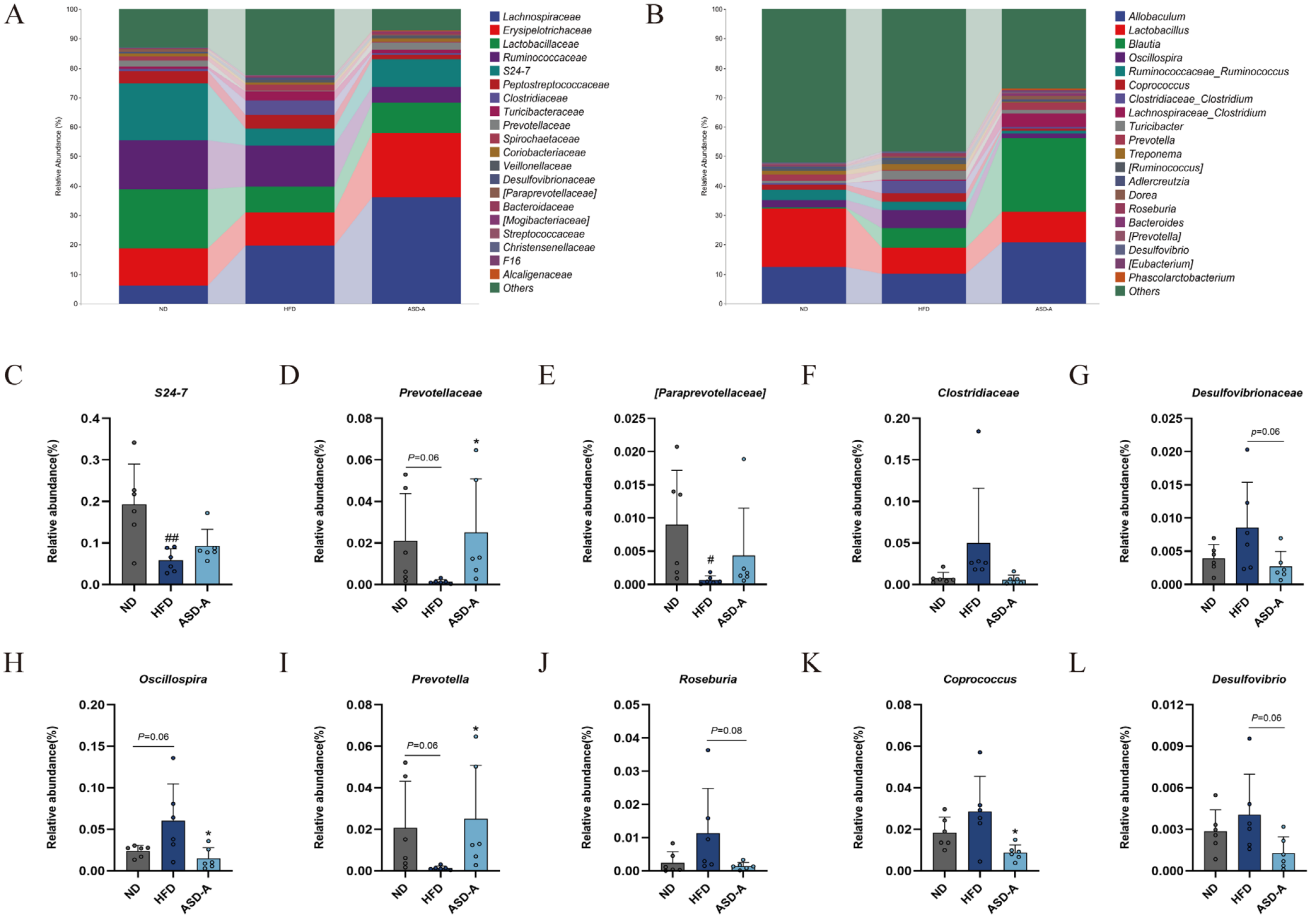
Changes in gut microbiota at the family and genus level in the ND, HFD, and ASD‐A groups (*n* = 6). (A) The relative abundance of species at the family (top 20); (B) The relative abundance of species at the genus level (top 20); (C–G) The relative abundance of *S24‐7* (*Muribaculaceae*), *Prevotellaceae*, [*paraprevollaceae*], *Clostridiaceae* and *Desulfovibrionaceae* at the family level; (H–L) The relative abundance of *Oscillospira*, *Roseburia*, *Prevotella*, *Clostridium* and *Coprococcus* at the genus level. Data are expressed as mean ± SD, ^#^ *p* < 0.05, ^##^ *p* < 0.01 compared with the ND group; *p < 0.05 compared with the HFD group.

Metabolic syndrome onset and progression often involve gut microbiota dysbiosis and dysfunction, which typically causes a reduction in beneficial bacteria, including *Prevotellaceae*, *S24‐7* (*Muribaculaceae*), and *Parabacteroides*, alongside an increase in pathogenic bacteria, including *Roseburia*, *Oscillospira*, and *Ruminiclostridium* (Lv et al. 2023; Wang et al. 2020). In the present study, at the family level (Figure 8A,C–G), the relative abundances of *Clostridiaceae* and *Desulfovibrionaceae* were significantly higher in the HFD group than in the ND group. In addition, the relative abundance of *S24‐7* (*Muribaculaceae*), *Prevotellaceae*, and [*paraprevollaceae*] considerably decreased in the HFD group. *Muribaculaceae* (S24‐7), which belongs to the phylum *Bacteroidetes*, was found to be lower in HFD‐fed mice (Lagkouvardos et al. 2019; Wu et al. 2023). Moreover, *Muribaculaceae* was reported to be associated with intact function of the inner mucus layer in the colon (Paone et al. 2022), which may be responsible for HFD‐reduced damage of the intestinal barrier (Figure 5). ASD‐A administration resulted in a markable increase in the relative abundance of *Prevotellaceae* (*p <* 0.05), with [*paraprevollaceae*] and *Muribaculaceae* showing an upward trend. Conversely, the relative abundance of *Clostridiaceae* and *Desulfovibrionaceae* revealed a declining tendency in the ASD‐A group. At the genus level, the relative abundance of *Roseburia*, *Oscillospira*, *Clostridium*, and *Coprococcus* in the HFD group were significantly increased, whereas the relative abundance of *Prevotella* decreased substantially. The relative abundance of *Oscillospira* and *Coprococcus* in the ASD‐A group decreased remarkably (*p <* 0.05), with declining trends in the relative abundance of *Clostridium* and *Roseburia*. In addition, the relative abundance of *Prevotella* was significantly increased (*p* < 0.05). In summary, *Prevotellaceae*, *Muribaculaceae, Prevotella*, and *Oscillospira* may be regarded as potential biomarkers for modulating the gut microbiota to prevent and alleviate metabolic syndrome.

### 3.7 ASD‐A Promotes the Production of SCFAs and BAs in HFD‐Fed Rats

**FIGURE 9:**
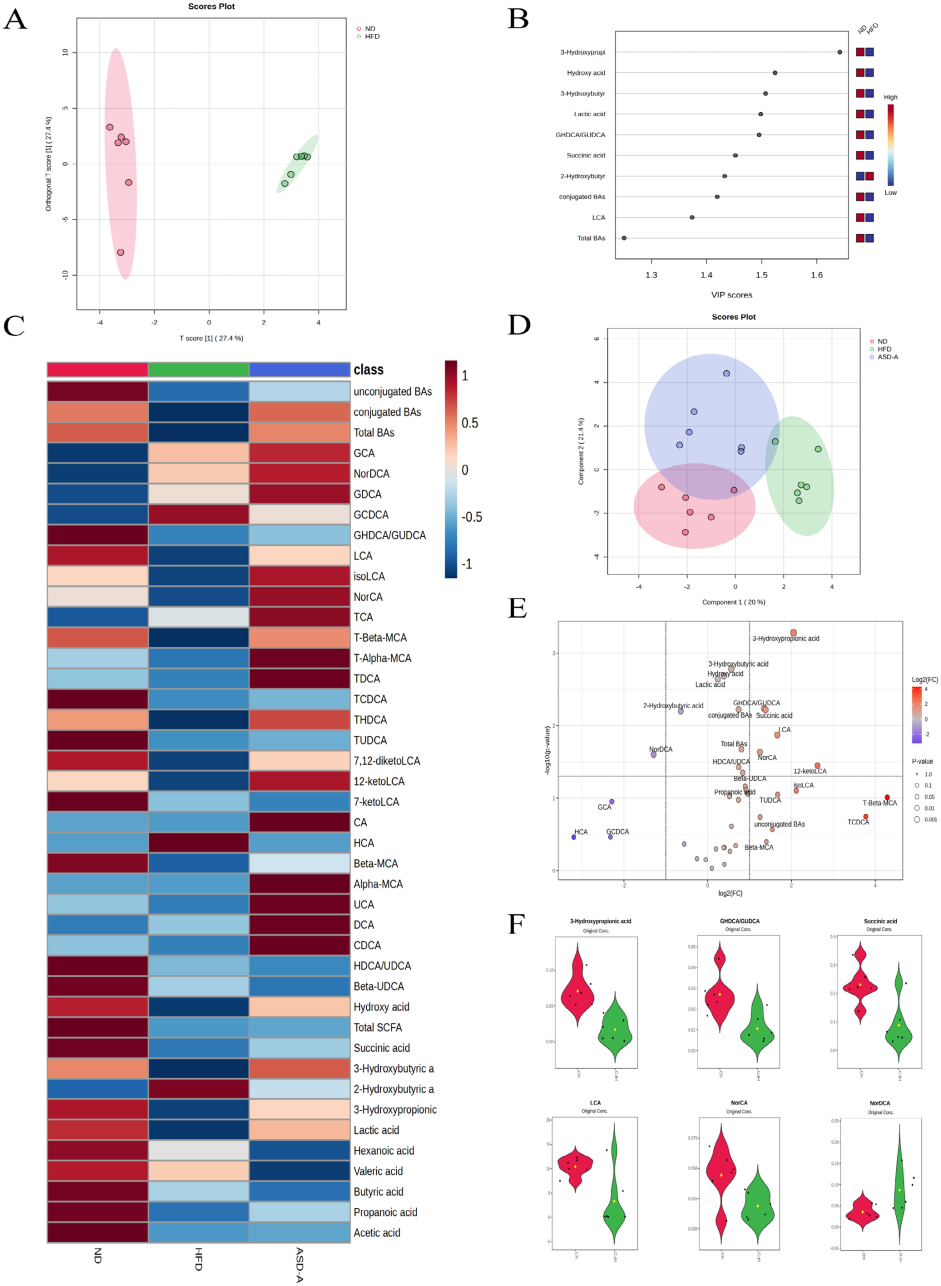
Effects of ASD‐A on SCFAs and BAs contents in rat feces. (A) OPLS‐DA plot among ND and HFD groups; (B) VIP plot based on OPLS‐DA plot among ND and HFD groups; (C) Heatmap analysis on metabolites in rat feces; (D) SPLS‐DA plot among ND, HFD and ASD‐A groups. (E) Volcano plot of differential metabolites of the ND vs. HFD groups (FC > 2, *p* < 0.05); (F) Violin plot of differential metabolites based on Volcano plot.

The bidirectional regulation of gut microbiota and metabolites, such as SCFAs and BAs, plays a crucial role in metabolic diseases. Gut microbiota dysbiosis usually results in a decrease in SCFA and BA levels, while SCFA or BAs supplementation may improve metabolic diseases such as liver disease and diabetes (Hong et al. 2021). In this study, HPLC‐MS based targeted metabolomics methods were employed to carry out a more in‐depth analysis of SCFAs and BAs in rat fecal samples, which were obtained from ND, HFD, and ASD‐A groups. Differential metabolites analyses were firstly carried out between ND and HFD groups. The results, as depicted in Figure 9A, revealed a distinct separation, indicating compositional differences in the OPLS‐DA between the two groups. The VIP score was utilized to further identify differential metabolites, ranking among the top: 3‐HP, 3‐HB, LA, GASCA/GUDCA, SA, 2‐HB, and LCA (Figure 9B). The volcanic plot analysis identified six upregulated differential metabolites: 3‐HP, SA, NorCA, LCA, 12‐ketoLCA and GASCA/GUDCA, with one downregulated metabolite: NorDCA (Figure 9E,F). The effect of ASD‐A on metabolites related to the gut microbiota was further analyzed. The heatmap and SPLS‐DA analysis demonstrated that a compositional difference in metabolites among ND, HFD and ASD‐A groups (Figure 9C,D).

**FIGURE 10:**
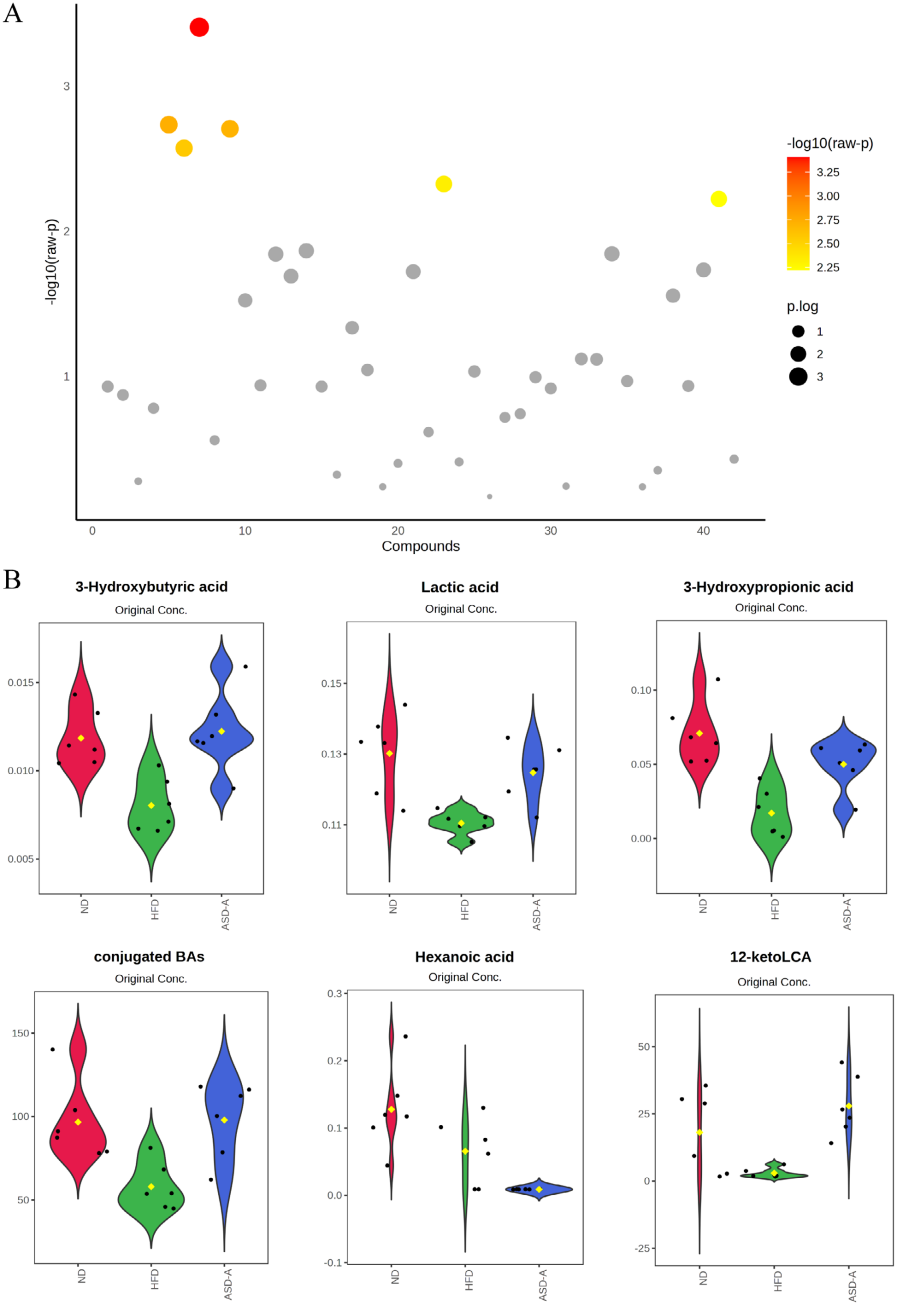
Effects of ASD‐A on SCFAs and BAs contents in rat feces. (A) ANOVA analysis among ND, HFD, and ASD‐A groups; (B) Violin plot of differential metabolites based on ANOVA analysis.

Based on the ANOVA (Figure 10A,B), six differential metabolites of 3‐HB, LA, 3‐HP, conjugated bile acid, HA, and 12‐ketoLCA were identified. ASD‐A showed a significant up‐regulation effect on HFD‐induced down‐regulation of 3‐HB, LA, 12‐ketoLCA, and conjugated bile acid. These findings indicated that ASD‐A regulated the production of certain metabolites, which may further affect the progress of HFD‐induced metabolic syndrome.

### 3.8 Spearman Correlation Analysis Revealed Potential Biomarkers for ASD Treatment of Metabolic Syndrome

**FIGURE 11:**
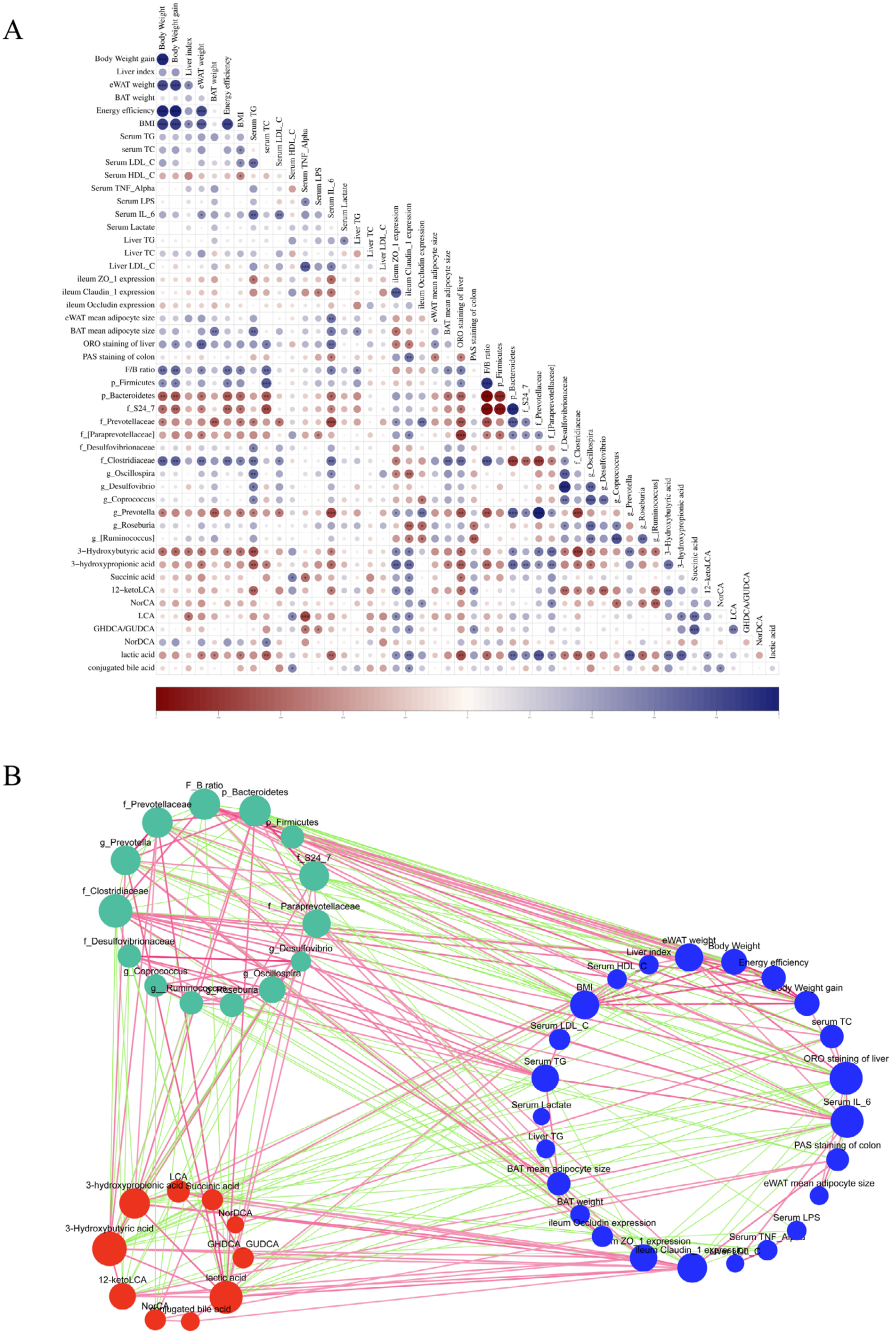
Spearman correlation network diagram among target metabolites, target gut microbiota, and clinical parameters of ASD treatment on metabolic syndrome. (A) Spearman correlation diagram, red represents negative correlation, blue represents positive correlation, “*” represents *p* < 0.05; (B) Spearman correlation network diagram, the green circle represents the target bacteria of ASD, the red circle represents the target metabolites of ASD, and the blue circle represents the clinical parameters of ASD treatment on metabolic syndrome. The green line represents a negative correlation, the red line represents a positive correlation, and the darker the color, the higher the degree of correlation.

Spearman correlation analysis was then conducted to further explore the relationship among metabolic syndrome‐related indicators, gut microbiota, and microbiota‐related metabolites. As shown in Figure 11A,B, F/B and *Firmicutes* had significant positive correlations with body weight, weight gain, epididymal fat mass, energy efficiency, BMI, serum TC levels, and other clinical indicators of metabolic syndrome; *Clostridiaceae* was positively associated with the above indicators and serum TG, LDL‐C, and IL‐6 levels. Conversely, *Bacteroidetes* and *Muribaculaceae* were significantly negatively correlated with body weight, weight gain, epididymal fat mass, energy efficiency, BMI, serum TC, and IL‐6 levels. *Prevotellaceae* had significant negative correlations with body weight, brown fat mass, BMI, serum TG, TC, LDL‐C, and IL‐6 levels. The correlations between gut microbiota‐related metabolites and clinically relevant indicators of metabolic syndrome were further analyzed. There was a significantly negative correlation between lactic acid and clinical indicators of metabolic syndrome, including eWAT and BAT mass, BMI, serum TG, and IL‐6 levels. 3‐HB had considerable negative correlations with body weight, weight gain, liver index, eWAT mass, energy efficiency, BMI, serum TG, and IL‐6 levels. 3‐HP exhibited a significant negative correlation with body weight, eWAT mass, and serum TG, TC, and IL‐6 levels. Notably, lactic acid, 3‐HB, and 3‐HP were significantly positively correlated with the expression of ZO‐1 and Claudin‐1 protein; *Bacteroidetes, Muribaculaceae*, *Prevotellaceae*, and *[Paraprevollaceae]*, while significantly negatively correlated with *Clostridiaceae* and *Oscillospira*.

The findings indicate that the gut microbiota and its metabolites have a regulatory influence on the clinical indicators of metabolic syndrome, which include body weight, blood lipid levels, inflammation, and the expression of intestinal barrier proteins. Notably, lactic acid, 3‐HB, and 3‐HP not only exhibited negative correlations with these clinical indicators but also positive correlations with the abundance of beneficial bacteria. Consequently, the content of lactic acid, 3‐HB, and 3‐HP is anticipated to serve as potential small‐molecule targets for ASD for treating metabolic syndrome.

## 4 Conclusion

Owing to their diverse bioactive properties, an increasing number of Chinese herbal medicines are playing an essential role as food supplements in promoting health and preventing diseases (Hossain et al. 2025). *Lycium chinense* and hawthorn are traditional medicines and dietary supplements used in China, respectively. The active component betaine in *Lycium chinense* promotes muscle differentiation and energy metabolism, enhancing muscular endurance in mice and myogenesis in myoblasts (Lee et al. 2021). Hawthorn seeds contain multiple active phenolic compounds that exhibit anti‐inflammatory and antioxidant effects (Żurek et al. 2024). *Platycodon grandiflorus* is a widely utilized edible medicinal herb with diverse biological applications, including hypotensive, hypolipidemic, anti‐atherosclerotic, anti‐inflammatory, antitussive, and expectorant effects, promotion of bile acid secretion, and antioxidant properties (Ji et al. 2020). In the present work, we conducted a systematic examination of the ameliorative effects of ASDs at three distinct pairing ratios on HFD‐induced metabolic syndrome. The present findings indicate that ASD‐A, with a quality ratio of 1:2 (AM: SM), demonstrated superior improvement in lipid metabolic disorders and attenuation of liver and intestinal barrier damage. In this process, the impact of ASD‐A on gut microbiota and its related metabolite (SCFAs and BAs) levels was analyzed. The results showed that ASD‐A effectively improved HFD‐induced gut microbiota disorders and enhanced metabolite production. Dietary supplements and functional foods are increasingly recognized as adjunctive therapeutic options. Astragalus membranaceus and *Salvia miltiorrhiza* are representative traditional Chinese medicinal herbs with multiple pharmacological activities, including anti‐inflammatory, antioxidant, and neuroprotective effects. In recent years, a growing body of research has incorporated Astragalus membranaceus and *Salvia miltiorrhiza* into dietary supplements for populations undergoing disease treatment (Meng et al. 2025; Ny et al. 2021; Shen et al. 2023). The findings of this study may promote the potential development of Astragalus membranaceus and *Salvia miltiorrhiza* combinations as functional foods and dietary supplements for preventing and improving metabolic syndrome.

## Author Contributions

**Haiyin Zhang:** investigation, Data curation, Writing – original draft. **Haofang Wan:** investigation, Formal analysis, Methodology, Writing – original draft. **Yihang Lu:** investigation, Validation, Writing – original draft. **Yu He:** methodology, Writing – review and editing. **Haitong Wan:** resources, Supervision, Writing – review and editing. **Chang Li:** conceptualization, Funding acquisition, Writing – review and editing.

## Funding

This study supported from National Natural Science Foundation of China [82174269], Zhejiang Traditional Chinese Medicine Administration [GZY‐KJS‐ZJ‐2025‐035] are gratefully acknowledged.

## Conflicts of Interest

The authors declare no conflicts of interest.

## Supporting information


**Table S1:** Tandem MS parameters for SCFAs and internal standards.
**Table S2:** Mass spectrometry information for BAs and internal standards.
**Table S3:** Standard curve information of the components obtained in ASDs.

## Data Availability

The data that support the findings of this study are available from the corresponding author upon reasonable request.
