# Supplementary material for: Astragalus membranaceus—Salvia miltiorrhiza Decoction Ameliorates Metabolic Syndrome and Gut Microbiota Dysbiosis Induced by High‐Fat Diet: A Comparative Study
Source: Food Sci Nutr. 2026 Jan 9;14(1):e71436. doi: 10.1002/fsn3.71436 (PMC12784168; doi:10.1002/fsn3.71436)
Supplement: Supplementary file 1 — Table S1: Tandem MS parameters for SCFAs and internal standards. Table S2: Mass spectrometry information for BAs and internal standards. Table S3: Standard curve information of the components obtained in ASDs. [file FSN3-14-e71436-s001.docx]

***Astragalus membranaceus - Salvia miltiorrhiza* decoction ameliorates metabolic syndrome and gut microbiota dysbiosis induced by high-fat diet: A comparative study**

***Supplementary Materials***

| **Table of contents** | **Page** |
| --- | --- |
| Derivation and determination of fecal levels of SCFAs | 2 |
| Determination of fecal levels of BAs | 3 |
| Gut microbiota analysis | 4 |
| **Table S1.** Tandem MS parameters for SCFAs and internal standards. | 6 |
| **Table S2.** Mass spectrometry information for BAs and internal standards | 7 |
| **Table S3.** Standard curve information of the components obtained in ASDs | 8 |

**Derivation and determination of fecal levels of SCFAs**

**Internal standard solutions.** 0.2mg/L of propionic acid-D_5_ (PA-d_5_), succinic-D_4_ acid (SA-d_4_) and Hexanoic acid-D_11_ (HA-d_11_), 1mg/L of L-Lactic acid-^13^C_3_ Sodium (L-LA-^13^C_3_).

**Derivation process.** 50 mg of lyophilized fecal sample was precisely weighed, extracted with 50 % methanol with ultrasonication at 50 °C for 30 min, and centrifuged at 15000 g for 10 min. The supernatant (30 μL) was mixed with 0.3 M 4-bromo-N-methylbenzylamine (4-BNMA, 10 μL), 0.3 M 1-ethyl-3-dimethylaminopropyl carbodiimide (EDC, 10 μL), and mixed internal standard solutions (10 μL), followed by ultrasonication at 60 °C for 60 min. The mixture was carefully transferred to sample vials for LC/MS analysis.

**HPLC conditions.** SCFA derivatives were separated using an Ultimate XB-C18 column (150 mm×4 mm, 3μm, Shanghai, China). The mobile phases consisted of a 0.1% formic acid aqueous solution (phase A) and methanol–acetonitrile (1:1, containing 0.1% formic acid, phase B). The gradient elution program was as follows: 0 min, 50% B; 5 min, 70% B; 20 min, 80% B; 22 min, 95% B; 35 min, 95% B. The flow rate was maintained at 0.3 mL/min, with an injection volume of 2 μL.

**Mass spectrometry parameters.** Mass spectrometry was conducted under the following conditions: Curtain Gas, 30 Psi; Ion Spray Voltage, 4500 V; Temperature, 500 ℃；Ion Source Gas1, 50 Psi; Ion Source Gas2, 50 Psi. The scan was performed under positive MRM mode.

**Determination of fecal levels of BAs**

After collection, the fecal samples were lyophilized and extracted with methanol in an ultrasonic bath at 60 ℃ for 30 min, followed by centrifugation at 12,000 rpm for 10 min. The resulted solution dried under nitrogen gas. Thereafter, the extracted sample was re-constituted in 50% methanol solution, in which internal standard was added. The obtained solution was then analyzed with LC-Q-Exactive MS. The chromatographic separation was achieved using a YILITE Supersil ODS2 column (4.6 mm × 150 mm i.d., 3 µm, Yilite) with a mobile phase flow rate of 0.6 mL/min. The mobile phases employed were 0.1% formic acid aqueous solution (phase A) and acetonitrile (phase B). The gradient elution program was as follows: 0 min, 20% B; 10 min, 60% B; 20 min, 75% B; 25 min, 95% B; 30 min, 95% B. The flow rate was set at 0.6 mL/min, and the injection volume was 20 µL. The mass spectrometry conditions comprised the following: sheath gas, 50 psi; auxiliary gas, 14 psi; sweep gas, 3 psi; temperature of the capillary, 300 ℃; temperature of the auxiliary gas, 500 ℃; spray voltage, 2.5 kV; automatic gain control goal (ACG), 2×104; maximum injection time, 200 ms; resolution, 70,000. The quantitative analysis was performed by the peak area ratio of BAs to IS, and the quantitative data were processed by Xcalibur software (Thermo, v4.1).

**Gut microbiota analysis**

Total fecal microbial genome DNA was extracted by CTAB method. DNA concentration and purity were monitored on 1% agarose gel. The DNA was diluted to 1 ng/μL with sterile water.

PCR amplification of target fragment, forward primer 515F (5'-GTGCCAGCMGCCGCGGTAA-3') and reverse primer 806R (5'-GGACTACHVGGGTWTCTAAT-3') were used for PCR amplification of V3-V4 regions of bacterial 16S rRNA gene.

The mixture of PCR products was purified using the Qiagen Gel Extraction Kit (Qiagen, Germantown, USA). The TruSeq® DNA PCR-Free Library Construction Kit (Illumina, San Diego, USA) was used to construct the sequencing library, and the index code was added. Finally, 250 bp sequencing of paired ends was performed using Illumina Novaseq platform.

FLASH (Version 1.2.7) was used to splice reads of each sample to get raw tags, and the QIIME (Version 1.7.0) quality control process was used to get clean tags. Using the UCHIME algorithm (UCHIME Algorithm) to connect the tag with the reference database (Silva database) for comparison to detect chimera sequences, which were removed to obtain effective tags. Sequence analysis was performed by Uparse software (Uparse v7.0.1001). Sequences with ≥97% similarity were assigned to the same OTUs. For each representative sequence, the Silva Database was used to annotate the classification information based on the Mothur algorithm. In order to study the phylogenetic relationship between different OTUs and the differences of dominant species in different samples (groups), multiple sequence allocations were performed by Marken software (Version 3.8.31). OTUs abundance information was standardized using a standard sequence number corresponding to the sample with the least number of sequences. The α diversity was used to analyze the complexity of the species diversity in the sample, including observed species, chao 1, Shannon, Simpson, ACE, and good coverage, calculated by QIIME (Version 1.7.0) and displayed by R software (Version 2.15.3). The beta diversity of weighted and unweighted homogeneity was calculated by the QIIME software (Version 1.9.1). Principal component analysis (PCA) and Principal co-ordinates analysis (PCoA) analysis were shown in the R software (Version 2.15.3) using the WGCNA package, stat package, and ggplot2 package. Unweighted Pair-group Method with Arithmetic Means (UPGMA) Clustering was performed as a type of hierarchical clustering method to interpret the distance matrix using average linkage and was conducted by QIIME software (Version 1.9.1). Linear discriminant analysis (LDA) coupled with effect size (LEfSe) was performed to identify the bacterial taxa differentially represented between groups at the genus or higher taxonomic levels, and was conducted by LEfSe software (LEfSe 1.0). The functional profiles of microbial communities were predicted by using PICRUSt. OTUs were picked using a closed reference (Greengenes ver. 13.5) at 97% sequence similarity, with normalization to control for differences in 16S rRNA copy number among OTUs. The relevant predicted genes and their function were aligned to KEGG database and the differences among groups were compared with the STAMP software (http://kiwi.cs.dal.ca/Software/STAMP). The two-side Welch’s t-test and Benjamini-Hochberg FDR correction were used in the between-group analysis. ANOVA with the Dunnett-Kramer test with the Benjamini-Hochberg correction were chosen for multiple-group analysis.

**Table S1.** Tandem MS parameters for SCFAs and internal standards.

| Analyte | MRM transitions | DP (v) | CE(v) | CXP(v) |
| --- | --- | --- | --- | --- |
| AA | 242/169 | 80 | 19 | 6 |
| PA | 256/169 | 80 | 35 | 12 |
| BA | 270/169 | 80 | 35 | 6 |
| VA | 284/169 | 80 | 25 | 8 |
| HA | 298/169 | 80 | 41 | 41 |
| LA | 272/169 | 80 | 21 | 7 |
| 3-HP | 272/169 | 80 | 21 | 7 |
| 2-HB | 286/169 | 80 | 50 | 13 |
| 3-HB | 286/169 | 80 | 50 | 13 |
| SA | 482/169 | 80 | 49 | 16 |
| PA-d_5_ | 261/169 | 80 | 35 | 12 |
| HA-d_11_ | 309/169 | 80 | 41 | 14 |
| L-LA-^13^C_3_ | 275/169 | 80 | 50 | 7 |
| SA-d_4_ | 486/169 | 80 | 49 | 16 |

CE, collision energy; CXP, collision cell exit potential; DP, declustering potential.

**Table S2.** Mass spectrometry information for BAs and internal standards.

| Analyte | Mass[m/z] | Formula[M] | Ion Type | CS[z] |
| --- | --- | --- | --- | --- |
| CA, β-MCA, α-MCA, UCA, HCA | 407.2803 | C_24_H_40_O_5_ | [M-H]^-^ | 1 |
| LCA, isoLCA | 375.2905 | C_24_H_40_O_3_ | [M-H]^-^ | 1 |
| DHA | 401.2334 | C_24_H_34_O_5_ | [M-H]^-^ | 1 |
| CDCA, HDCA, DCA, UDCA, β-UDCA | 391.2854 | C_24_H_40_O_4_ | [M-H]^-^ | 1 |
| GCA | 464.3018 | C_26_H_43_NO_6_ | [M-H]^-^ | 1 |
| Glycyrrhetic Acid (IS) | 469.3323 | C_30_H_46_O_4_ | [M-H]^-^ | 1 |
| TCA, T-β-MCA, T-α-MCA | 514.2844 | C_26_H_45_NO_7_S | [M-H]^-^ | 1 |
| TDCA, TCDCA, THDCA, TUDCA | 498.2895 | C_26_H_45_NO_6_S | [M-H]^-^ | 1 |
| 7-ketoLCA, 12-ketoLCA | 389.2697 | C_24_H_38_O_4_ | [M-H]^-^ | 1 |
| GDCA, GUDCA, GCDCA, GHDCA | 448.3069 | C_26_H_43_NO_5_ | [M-H]^-^ | 1 |
| NorDCA | 377.2697 | C_23_H_38_O_4_ | [M-H]^-^ | 1 |
| TLCA | 482.2946 | C_26_H_45_NO_5_S | [M-H]^-^ | 1 |
| GLCA | 432.3119 | C_26_H_43_NO_4_ | [M-H]^-^ | 1 |
| 7,12-diketoLCA | 403.2490 | C_24_H_36_O_5_ | [M-H]^-^ | 1 |
| NorCA | 393.2647 | C_23_H_38_O_5_ | [M-H]^-^ | 1 |

**Table S3.** Standard curve information of the components obtained in ASDs

| components | standard curve | R^2^ | linear range（mg/L） |
| --- | --- | --- | --- |
| salvianolic acid B | y = 19.831x + 3.6831 | 0.9999 | 50-450 |
| calycosin-7-O-β-D-glucoside | y = 48.293x - 0.5467 | 0.9997 | 0.25-2.5 |
| ononin | y = 25.733x + 5.8817 | 0.9981 | 1-10 |
